# Whole-Genome Sequencing of *Xanthomonadaceae* Strain Alg18-2.2, Isolated from the Saline Lake Gudzhirganskoe in the Republic of Buryatia, Russia

**DOI:** 10.1128/MRA.01112-19

**Published:** 2019-11-14

**Authors:** Andrey V. Karlyshev, Ekaterina B. Kudryashova, Elena V. Ariskina, Elena Y. Abidueva, Elena V. Lavrentyeva, Darima D. Barkhutova

**Affiliations:** a School of Life Sciences, Pharmacy and Chemistry, SEC Faculty, Kingston University, Kingston upon Thames, United Kingdom; b G. K. Skryabin Institute of Biochemistry and Physiology of Microorganisms, Pushchino Scientific Center for Biological Research of the Russian Academy of Sciences, Russian Academy of Sciences, Pushchino, Russia; c Institute of General and Experimental Biology, Siberian Branch Russian Academy of Sciences, Ulan-Ude, Russia; University of Southern California

## Abstract

A draft genome sequence of the bacterial isolate Alg18-2.2, recovered from the highly saline and alkaline lake Gudzhirganskoe (Buryatia, Russia), was determined. The results of bacterial identification using 16S rRNA gene sequence and whole-genome analyses suggest that the bacterium belongs to a novel genus. Some genomic features are discussed here.

## ANNOUNCEMENT

Strain Alg18-2.2 (VKM B-3394) was isolated on 7-fold-diluted #1 GRM medium (Obolensk, Russia) with 5% sea salt and 1.5% agar (pH 9.5) from the bottom sediment sample of the highly alkaline (pH 9.4) and saline (26 g/liter) lake Gudzhirganskoe (Buryatia, Russia), as described previously (1). The lake belongs to the algal group of lakes, which are a subject of intense research (1). Microorganisms living under such extreme environmental conditions have exceptional adaptive physiological ability and are of special interest due to their great biotechnology potential (2).

DNA was extracted from bacteria grown in tryptone soya broth with 5% sea salt (pH 9.5) at 28°C using a total DNA extraction kit (Yeast/Bact. kit B; Qiagen). The sequencing library was constructed using the NEB fast fragmentation and library preparation kit (New England BioLabs). The 480- to 500-bp adapter-ligated DNA fragments were PCR amplified with primers 5′-CCATCTCATCCCTGCGTGTC (forward) and 5′- CCACTACGCCTCCGCTTTCCTCTCTATG (reverse) and analyzed using a high-sensitivity DNA kit and BioAnalyzer 2100 (Agilent). The template was prepared using the Ion Torrent OneTouch system and Ion Personal Genome Machine (PGM) Hi-Q View OT2 kit. The sequencing reaction was conducted using the Ion Torrent PGM, a 316v2 Chip, and the Ion PGM Hi-Q View sequencing kit. This generated 423,474 single-end reads with a mean size of 313 bases and a total of 132,604,596 bases. Default parameters were used for all software, unless otherwise specified. The sequencing reads were assembled *de novo* using the Torrent SPAdes plugin 5.0.0.0 into 20 contigs (maximum size, 613,806 bases), with 43.64× coverage, an *N*_50_ value of 256,677 bases, and a total assembly size of 3,025,752 bases with 68.7% G+C content.

Initial bacterial identification of the strain was performed via analysis of its complete 16S rRNA sequence (1,540 bases) derived from the genome assembly. A sequence similarity search using the NCBI BLASTN server and bacterial 16S rRNA gene sequence database produced the best hit with the 16S rRNA gene sequence of Lysobacter spongiicola (NCBI RefSeq accession number NR_041587), with 94.80% identity and 100% coverage. The top hits generated by the whole-genome bacterial identification tool EZBioCloud (3) were also with the genomes of *Lysobacter* spp. However, the level of 16S rRNA gene sequence similarity, as well as the average nucleotide identity (ANI) percentage and coverage (up to 83.53% and 12.6%, respectively) were too low to assign our strain to the Lysobacter genus. It is likely that the strain represents a novel genus of the *Xanthomonadaceae* family.

**FIG 1:**
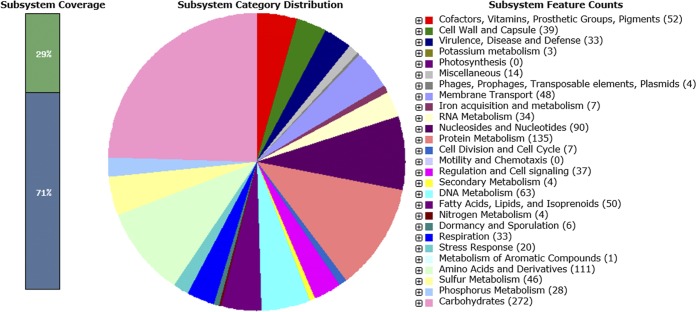
Subsystem category distribution of genes found in strain Alg18-2.2, according to genome sequencing data analysis using the RAST server (5).

The draft genome sequence was annotated using the NCBI PGAP (4) and Rapid Annotations using Subsystems Technology (RAST) (5) tools. The NCBI GenBank annotation records were produced using the PGAP. According to the results generated using the RAST tool (Fig. 1), only type IV and VI protein secretion systems and one toxin-antitoxin system were present, and resistance to only one class of antibiotics (fluoroquinolones) was predicted. As expected from the bacterium’s ability to survive in a highly saline environment, we found genes involved in the synthesis of osmoregulated periplasmic glucans, as well as oxidative stress-related genes.

The genomic features identified in strain Alg18-2.2 will help in taxonomy and an understanding of the mechanisms of bacterial survival under extreme physiological conditions.

### Data availability.

This whole-genome shotgun project has been deposited at DDBJ/ENA/GenBank under the accession number VRTS00000000. The version described in this paper is version VRTS01000000. Raw data are deposited in GenBank under SRA number SRR10038466.
